# Clinical Symptoms and Risk Factors in Older Adults with Limbic‐Predominant Age‐Related TDP‐43 Neuropathology in the Context of Mixed Dementia

**DOI:** 10.1002/alz.089663

**Published:** 2025-01-03

**Authors:** Imogene M Scott, Yara Alkhodair, Danielle Weber‐Adrian, Ian R MacKenzie, Ging‐Yuek Robin Hsiung

**Affiliations:** ^1^ The University of British Columbia, Vancouver, BC Canada

## Abstract

**Background:**

Limbic‐predominant age‐related TDP‐43 encephalopathy neuropathological change (LATE‐NC) is a pathological process diagnosed at autopsy, involving deposition of TDP‐43 in the medial temporal lobes. The name LATE‐NC was recently proposed to represent the pathological process, while “LATE” has been suggested to represent the clinical syndrome. However, there are currently no available criteria to diagnose this syndrome during life, and the clinical phenotype is not well understood.

**Method:**

A sample of autopsy cases (*n* = 39) from the University of British Columbia Hospital with moderate to severe Alzheimer and/or Lewy Body pathology, with and without LATE‐NC, underwent chart review. 19 individuals with LATE‐NC pathology (LATE+) were age‐matched to 20 controls (LATE‐) by age and by degree of Alzheimer and Lewy Body pathology. Clinical characteristics including ages at symptom onset, diagnosis and death; cognitive test scores; medical history and functional status were extracted from the clinical charts, and compared using the t‐test and χ^2^ statistics.

**Result:**

In the two groups, mean age at death was 80.7 years (LATE+) and 79.7 years (LATE‐). Age at onset (66.4 versus 68.4 years) and disease duration (11.9 versus 11.2 years) were not significantly different. Years of education (14.9 versus 13.3, *p* = 0.03), and prior history of head injury (33.3% vs 5.3%, *p* = 0.04) were both significantly higher in the LATE+ group. There was a trend towards a higher prevalence of mood symptoms in the LATE+ group (55.6% versus 36.8%), and fewer psychotic symptoms (10.5% versus 36.8%), however these differences were not statistically significant. There were no significant differences in cognitive domains affected across the disease course, or baseline cognitive test scores.

**Conclusion:**

In this study, head injury and greater years of education were identified as possible risk factors for LATE‐NC. There were differences in the burden of neuropsychiatric symptoms between the two groups which require further investigation. However, the presence of LATE‐NC did not appear to accelerate disease progression, suggesting its clinical impact is small relative to the impact of severe Alzheimer or Lewy Body pathology. Additional study including those with little or no concomitant pathology may help to elucidate the clinical phenotype further.

